# The Relationship Between Critical Social Theory and Interpretive Description in Nursing Research

**DOI:** 10.1177/23333936231211462

**Published:** 2023-11-24

**Authors:** Jane McCall, J. Craig Phillips, Andrew Estefan, Vera Caine

**Affiliations:** 1 University of Victoria, Victoria, BC, Canada; 2 University of Ottawa, Ottawa, ON, Canada; 3 University of Calgary, Calgary, AB, Canada

**Keywords:** interpretative description, critical social theory, harm reduction, substance use, addiction, opiate assisted treatment, Canada

## Abstract

This paper is an examination of the methodological and theoretical perspectives of a study with an inquiry focus on the experiences and perspectives of staff who worked at an injectable opiate assisted (iOAT) clinic. Twenty-two staff members, including nurses, social workers, and peer support workers, were interviewed. The goal of the study was to uncover how the clinic staff provided care to the clients who attend the clinic, their perspectives on how the clinic program impacted both them and their clients, and their experiences with the program itself. This interpretive descriptive study was underpinned by critical social theory. Thematic analysis was undertaken to identify recurring, converging, and contradictory patterns of interaction, key concepts and emerging themes. In this paper we examine and discuss how the relationship between critical social theory and interpretive description enhanced the study. Examples from the study are presented to provide insight into the relationship.

Researchers draw on theoretical perspectives for the phenomena and methodology of their research, as well as make claims about knowledge. Theoretical foundations inform all aspects of a study and identifying these foundations is a crucial first step in the research process (Vinz & George, 2022). Often theoretical perspectives inform the choice of methodology, yet at times researchers do not explicitly understand how methodological choices are made or that a choice is being made at all (Liu & Harrell, 2015). In some instances, researchers make choices about methodology based on *hunches*, or a general sense or feeling about what will work well (Lloyd-Walker & Walker, 2015). As the uptake of qualitative methodologies continues to grow, researchers need to clearly consider theoretical perspectives; it is necessary to have a clear ontological, epistemological and axiological stance in order to defend a research approach (Lloyd-Walker & Walker, 2015). Problematic gaps between theoretical perspectives and methodology do little to enhance the credibility of research findings. It is necessary that clear connections between the problem or phenomenon being studied, the theories that link the problem or phenomenon to the research question, the methodology, and subsequent methods are demonstrated (Connelly, 2014).

In this paper we offer an example of thinking about the relationship between theory and methodology. We draw on experiences and knowledge gained while conducting a qualitative study examining the experiences of staff in an opiate assisted treatment clinic. This study formed the basis for the first author’s doctoral research (McCall, 2018) and was informed by a critical social theory (CST) perspective and an interpretive descriptive (ID) methodology.

## What is Critical Social Theory?

Critical social theory (CST) is an approach to exploring practices that stress the reflective assessments and critique of society and culture. It is used to explore and question discourse, or patterns and systems of talk, and the social world that is shaped, constrained, and revised through discourse and discursive practices (Lupton, 1992; Sumner & Danielson, 2007). Such an approach emphasizes that CST is a broad theoretical perspective that integrates a complex set of theories. These include: the theory of false consciousness, which demonstrates the ways in which the self-understandings of a group of people are false; the theory of crisis, which is an examination of a group’s dissatisfaction and how this threatens the social cohesion of society; the theory of education which allows individuals to derive some benefit from knowledge; and the theory of transformative action which details a plan of action for change (Manias & Street, 2000). An underlying standpoint of CST is that dialog is value laden and that norms are contextual and depend on the situation and participants. Critical theorists also make visible that, in the course of life and living, some people experience power and privilege while others occupy more marginal and disadvantaged identities and experiences (Sumner & Danielson). A CST approach seeks to actively encourages individuals to question prevailing norms. In other words, “[t]he goal is transformation from the constraints of unequal power relationships through self-reflection” (Manias & Street, 2000, p. 51).

CST offers a way of understanding the political and social agendas that shape, constrain and influence people’s lives. It is an important tool for uncovering the complexities of the social world and how discourses that operate in the social world construct people and institutions in particular ways and it is useful to examine processes of marginalization because CST orientates researchers to think about ways power operates as an organizing social dynamic. It can be used to examine power relationships to determine who benefits from power and who is marginalized and how the processes of marginalization shapes people, structures, institutions and practices. It also allows questions to be raised that explore what is taken for granted, what norms are unchallenged, what works and for whom (Sumner & Danielson, 2007). CST is useful because it allows people to identify and describe systemic suppressions that are at work in society as a whole and in systems that deliver health and social care.

CST arose from Marxism and was first defined in 1937 by Max Horkheimer of the Frankfurt School of Sociology (McLaughlin, 1999). In the 1960s, a new generation of theorists including Juergen Habermas, Michel Foucault, and Paulo Freire proposed that CST was a form of scientific inquiry that described distortions and constraints that impeded free, equal and unconstrained participation in society (Fulton, 1997). This theory gained momentum in resistance movements where various scholars spoke out against social conditions such as fascism, racism, exploitation of women and prejudice against various groups, including people of color and the LGBTQ community (Hall, 1999). CST is driven by the possibility for empowerment, even liberation, from oppressions (Sumner & Danielson, 2007). For this reason, it is the people most affected by marginalization or oppression who define the issues that marginalize them as opposed to outside experts. Research guided by CST in nursing offers an opportunity for meaningful engagement with people who are marginalized and the issues that affect them. While engagement is critical, it is important that the impact of research is also assessed. In other words, did researchers achieve any meaningful change for the marginalized group they were working with (O’Byrne, 2019)?

CST is especially important in the care of people who are marginalized because it can illuminate issues they face that might otherwise remain hidden or obscured by dominant discourse and social practice. Care providers work within and for systems that marginalize patients, as well as the systems often marginalize them and their practices. It is important for care providers to question experience that happens in the tense spaces between the system in which they work and the need to respond in helpful and useful ways to the patients for whom they care (Stevens & Hall, 1992). Care providers need to focus on patients’ everyday concerns and the specific problems they identify. While they attend to immediate concerns requiring care, they also need to reflect and inquire into oppressive conditions experienced by themselves and patients. It is important to understand how these conditions might have developed, and what they can do to alleviate them. A CST approach can help to critique existing conditions in an effort to enhance individual autonomy and responsibility (Wilson-Thomas, 1995).

Historically, within the healthcare system, some care providers, like nurses, have rarely held positions of power. In the primary research (McCall, 2018), on which this article is based, the majority of participants were nurses. Nurses often see themselves as an oppressed group, within a system that relies on them, yet rarely values their input in developing policies and structures that determine their practice. Nurses do not practice outside of discursive constraints. Nurses’ capacities to critically reflect and act are constrained by dominant discourse about who nurses are and what they do in practice. Social and structural factors in both the activities and organization of nursing work implicitly communicate to nurses that they have less value than other members of the health care team (Hart, 2015). In some cases, this means nurses are constrained from acting in ways that help address patients’ marginal experiences and circumstances. In other instances, nurses become stigmatized and marginalized in practice because they work with already stigmatized patient groups, such as people living with HIV or people who use substances (Phillips, 2009).

CST offers insights that can be helpful for researchers as they decide how to conduct their research and as they negotiate the research process with participants. By using CST as a theoretical perspective, researchers seek to understand the world through the perspectives of their participants and how these perspectives can contribute to useful social action. The goals of research informed by CST are to make sense of the meaning others attribute to the world (Cresswell, 2006), as well as to identify social conditions that shape those meanings and to create movement toward change based on meanings that are identified.

## What is Interpretive Description?

Interpretive description (ID) is a qualitative methodology that acknowledges the constructed and contextual nature of human experience. An ID methodology reveals patterns and commonalities whilst at the same time allowing for inevitable individual variations (Thorne et al., 2004). Thorne et al. (2004) observe that investigators are rarely satisfied with description on its own. They are looking for meanings and explanations that will yield application to practice. ID is especially useful for researchers who want to know the who, what and where of events and experiences (Sandelowski, 2010).

ID provides useful and understandable guidance for a coherent research design. ID orients the researcher toward what is happening in the clinical context and the generation of practice-relevant findings. ID can also draw attention to disciplinary biases and commitments (Hunt, 2009); it has been extensively used in nursing research. ID is grounded in the idea that research aims to impact practice and that there is a need to understand what we know and do not know on the basis of existing empirical evidence. It allows researchers to go beyond the self-evident—in other words, beyond assumed and established knowledge, to see what else might be there (Thorne, 2008). ID is designed to account for the clinical context of research in applied health disciplines. The explicit relationship between ID and clinical practice orients data analysis toward the development of findings that will assist health care professionals in their practice (Hunt, 2009).

The above emphasis on ID as a means to examine the constructed and contextual nature of everyday life reveals its fit with a CST perspective. Ocean et al. (2022) note that ID is methodologically suited to investigating real world questions and its fundamental tenets are “compatible with and complementary to both participatory and decolonizing work” (p. 2). ID provides a grounding for concepts and their linkages that become apparent when one attempts to locate the specific in the universal, the state within the process, and the subjectivity of experiences within the usual conventions that current health care contexts represent as the temporal and symbolic location for health and illness (Thorne et al., 2004). The result of ID is to present a “tentative truth claim” (Thorne et al., 2004, p. 7) about what is common within a clinical phenomenon. Such a claim can inform clinical reasoning, extend insight for decision-making, and allow clinicians to make sense of the variations that occur within the real world of clinical practice.

Three ontological and epistemological criteria underpin the design of ID: first, reality is constructed, complex and subjective and can only be studied holistically. Second, the inquirer and the participant interact to influence one another and meaning must be constructed through negotiations with research participants as opposed to the researcher imposing meaning on findings. Third, due to the probable emergence of multiple realities from the findings, theory must emerge from and be grounded in the data (Lincoln & Guba, 1985). This emergence and grounding allows for the creation of an interpretive account that is a result of informed questioning and critical examination. ID is anti-oppressive and emancipatory in that it allows for the airing of variations and diversities within data sets and the translation of participant’s voices into actionable knowledge (Ocean et al., 2022).

There are similarities between these philosophical criteria and CST. CST is sensitive to context and the complexities of the social world. As such, CST and ID both attend to subjective experiences that arise in complex social circumstances (Sumner & Danielson, 2007). CST is contingent on understanding the language of oppressed persons themselves rather than relying on outside “experts” to name individual and social problems or experiences (Stevens & Hall, 1992). Here CST and ID are aligned, in that ID research involves negotiating meaning with participants rather than imposing it (Stevens & Hall, 1992).

Thorne (2008) contended that ID, unlike most qualitative methodologies, is not reliant on a theoretical fore-structure, but instead, is located within a disciplinary orientation, which is appropriate when researching a clinical problem or population. One could however argue that a disciplinary orientation is a kind of theoretical fore-structure. Yet, in nursing, there has been a history of front line nurses feeling disconnected from the theoretical world (Wilson-Thomas, 1995). Hence, nurses at times experience a gap between the world of practice and the theory and research meant to guide it. At the same time, it is important to recognize that theory helps nurses to “understand, examine, illuminate and facilitate empowerment for people who are interacting with the health care system” (Wilson-Thomas, 1995, p. 572). ID is a methodology that is transparent, equitable and creditable (Ocean et al., 2022). ID and CST both point toward generating ways of thinking that become optimally relevant to the practice context of nurses. CST and ID, together, offer a perspective on research and practice that can help to narrow the divide between research-derived knowledge and the complex world of practice.

## Turning Toward the Study

In the study on which arguments about theory and methodology are made visible, we examine the experiences and perspectives of staff who worked at an iOAT clinic (McCall et al., 2019). The first author has extensive experience as a nurse educator with the clinic and sought to explore and understand what motivated the staff to work in what, on the surface, seems to be a challenging environment. While there are a number of clinics that provide opiate assisted treatment in Europe, this is currently the only clinic of its kind in North America.

As part of the study, 22 staff members were interviewed, including 18 nurses, two social workers and two clinic support workers. Staff experience at the clinic ranged from 6 months to more than 10 years. An open-ended questionnaire was used to guide conversations and to understand the perspectives, opinions and experiences of staff. Data analysis revealed six themes including from chaos to stability; it’s not all roses; the stigma hasn’t gone away; putting the patient at the center; the clinic is life transforming; and the patients have a story to tell. The themes are discussed below in relation to CST and ID. Ethical approval was obtained from the University of Alberta and the University of British Columbia. Participants approached the researcher and indicated their desire to participate in response to a poster that was displayed in the clinic. Consent was both verbal and written. Participants were given a gift card in acknowledgment of their participation (McCall, 2018).

## Putting it Together

CST is a philosophical position that works well in ID and it provides an overarching lens for this study. It is especially relevant when looking at the experiences of people who are marginalized (Sonn & Quayle, 2013). Although front-line staff in health care settings are not generally considered to be marginalized, the issues of powerlessness that front line staff experience have been identified (Brathwaite, 2018; Hart, 2015; Hutchinson & Jackson, 2015). The research questions that framed this study were specifically aimed at understanding the experiences of staff and explicating these experiences in a way that would highlight their issues. The questions were designed to elicit these experiences. The intent was to understand how the staff navigated a world that often did not allow them to practice unimpeded by the expectations of their managers, the policies that guided their practices, and the design of their workplace. There is a good fit with ID, which is looking for how the constructed and contextual nature of the workplace impacts health care practice. Taking a CST approach to ID allowed for a research design that encouraged negotiation of meaning beyond a descriptive level (Lather, 1986). It drew attention to how the constructed and contextual nature of the workplace influenced and shaped practice.

Because this was an ID study, shaped by CST, what was important was for meaning—derived from the analysis and interpretation of the data—to transform practice. Lather (1986) referred to the connection between theory and practice as praxis, and was most concerned with the development of emancipatory knowledge. This is similar to Friere (1970), who pointed out that people need to reflect and act upon the world in order to transform it. Freire believed that humans have a capacity for historical, cultural, and linguistic praxis and that people produce history and culture, even as history and culture produce them. People see their reality as presenting concrete problems as well as opportunities for transformation (Glass, 2001). The staff in this study clearly understood the problems of their workplace and the findings of this study gave them the impetus to seek emancipation and transformation (McCall, 2018).

## Interpreting the Findings

The interpretation of the findings makes visible how CST shaped understandings, while ID held open a space of exploring practices and experiences that were situated in complex social, political, and legal contexts.

### From Chaos to Stability

One of the study’s themes, from chaos to stability, described how the patients achieved stability as a result of being able to receive legal heroin in a medical setting. But it was not just about stability. As a result of being able to access heroin without fear of arrest, incarceration or public shaming, the patients found power: power to make decisions about their lives, and power to articulate and make their treatment objectives known to the staff. The issue of power is a central idea in CST. CST attempts to lay bare the social sources of oppression (Leonardo, 2004). ID, with its emphasis on translating data into actionable findings, allowed for the recognition of how care providers supported patients. In the case of the patients, providers revealed how they observed a change in patient’s social status because of being able to access legal heroin. Several participants commented on the changes they saw in the patients in relation to this and they attempted to assist their patients in making the most of their unfolding experiences of having power.

### It’s Not All Roses

The second theme, it’s not all roses, revealed that the clinic was challenged by space restrictions. Many of the participants talked about the challenges of working in an inadequate space. The staff were concerned about the space, which was prone to mold, water leaks, and overcrowding. In occupational contexts, larger or more spacious work spaces are linked to respect, seniority, authority, and influence. Space, thus, signals and confers power (Gieryn, 2000). Space is socially produced and constructed and it could be said that space “keeps people in their place” (Kitchin, 1998, p. 343). From a CST perspective, space is in a complex power relationship with agency. Space is fundamental to any exercise of power (Soja, 2003). A lack of space made it difficult for the staff to do their work in the way they wanted to do it. As Putnam (1999) suggested, “our words and our life are constrained by a reality not of our own invention” (p. 76). The staff at the clinic were constrained and the limitations posed by the space made it difficult for the staff to engage in reflective practice. All of the participants talked about the challenges of working in such a constrained environment. They were well aware that the limited space reflected their leadership’s attitudes about their value as employees.

The challenge to engage in reflective practice limited the staff’s ability to question how oppressive conditions have developed and what they would do to relieve those conditions. ID, with its emphasis on construct and context, illuminated these findings. Lack of or poorly designed space affects peoples’ ability to do their job (Kasule, 2015). A CST perspective indicates that the dominant reality imposed by the clinic’s design inhibits care provider’s ability to practice (Mantzoukas & Jasper, 2004).

### The Stigma Hasn’t Gone Away

Stigma is a social process that unfolds in social spaces (Yang et al., 2007). The experience of stigma is associated with CST in that stigma arises from and sustains existing social hierarchies (Poteat et al., 2013). It is both a product and an enactment of power. Participants talked at length about the experience of stigma, both for themselves and their patients. Participants had experienced a lack of understanding and support for their job choice from friends, family and acquaintances, and they reported that the stigma their patients experienced extended to them. Stigma has negative political, social, economic and psychological consequences (Crocker & Major, 1989). From a CST perspective, stigma is a marginalizing discourse that is a tool of oppression. Through the use of ID, the focus shifted beyond the immediacy of the practice site and also considered people’s realities outside of this context. Participants were invited to share experiences that crossed between their private lives and their practices as care providers.

### Putting the Patient at the Center

The staff exhibited their own critical perspective in the way that they embraced the concept of patient centered care. The way they described putting the patient at the center and giving them power over their health care decisions was compelling evidence of their drive to ensure their patients had autonomy and shared in the decision-making. ID is explicitly associated with clinical practice and the findings in this study made clear how care was delivered and provided rationale for it. The practitioners in this clinic may not be familiar with CST but they were nonetheless identifying the systemic oppression that is at work in a health care setting and taking steps to ensure that their patients did not have negative experiences as a result of this.

### The Clinic is Life Transforming

The staff at the clinic related that their work experiences were life transforming. They talked about how they had to “hear people out” and how their interactions called them to engage in advocacy work. They came to understand their clients’ experiences of trauma and how they had to bring a compassionate stance to their work. Adversity was also an issue for clients and staff. The staff often had to defend their choice of employment to the public at large. Overcoming this sense of adversity led to a sense of achievement in their work. Through the use of ID, conversations that called forth vulnerabilities were encouraged and supported through an ethical stance that centered on care and responsibility.

### The Patients Have a Story to Tell

When this study was originally proposed, the plan was to interview patients as well as staff. However, the REB made their stance clear from the outset. They were reluctant to give approval to interview patients because there were ethical concerns about consent from individuals under the influence of heroin. The participants were vehemently opposed to the REBs stance. They made it clear to the researcher that they felt that the patients had important contributions to make to the study.

Excluding vulnerable people due to concerns about inebriation has the unintended consequence of being discriminatory and denying them the opportunity to contribute to society through research (McCall et al., 2020). Responsible advocacy, with its emphasis on maximizing a vulnerable participants’ capacity for self-determination, is a central tenet of CST. A researcher who is a responsible advocate does not just look at the obvious issues that affect participants but attends to issues that contribute to social inequality (Hopkins et al., 2004; L. J. Smith, 2008).

## Reflections on CST and ID

Utilizing CST reveals the complex political and ideological agendas that influence people’s lives. In the context of this study, it sheds light on particular experiences that give us an understanding of the impact of stigma and power. When one looks at stigma and power through the lens of CST, complex issues of marginalization, oppression, racism and gender discrimination become apparent.

From a philosophical standpoint, ID assumes that it is impossible to attain objective knowledge through empirical analysis (Thorne, 2008). CST helps to uncover the complex political, ideological and social agendas that affect people’s lives. Realities are local, experientially based and contingent on the persons who hold them (Hunt, 2009). Using ID, spaces were created in interviews that allowed people to show their vulnerabilities and engage in the study in meaningful ways. The goal of ID is to inform clinical practice (Hunt, 2009), which in turn is reliant on an understanding of patients—where they come from and what they believe, as well as the experiences of health care providers. Care providers’ goals, and nursing’s goals specifically, in relation to patients and their social and moral mandates for practice are inherently emancipatory (Browne, 2000), yet they are not always acted upon. In this current study, the research provided knowledge that can leverage change and as such be emancipatory. As Thorne (1997) contended, an emancipatory perspective shifts human inquiry away from straightforward knowledge acquisition toward a domain of generating practical knowledge, disrupting the patterns of power and participating in socially transformative activities that lead toward justice, equity and freedom. However, practice cannot exist without theory. In fact,

What is required is a dialectical unity of theory and practice so that theory guides and informs practice and practice guides and informs theory. Practice without theory is mindless activity, but theory without practice is verbalism (Lantholf & Poehner, 2014, p. 203).

A CST approach helped bridge the gap between theory and practice through the process of reflection on practice (Manias & Street, 2000), which is also encouraged in ID. This process of reflection is the first step toward advocacy. CST not only accepts the reality of oppression, it also assumes the possibility of a less oppressive condition. For participants in this study, a less oppressive condition would be to have a voice that is heard and acted upon and to minimize the stigma that they experience as a result of their job choice. CST does not surrender the search for emancipation but qualifies it as an unending process of liberation and multiple emancipations (Leonardo, 2004). Taking a CST perspective means undertaking a conscious remaking of the world (Freire, 1998).

In the case of this study, taking a CST perspective led to a greater understanding of the forces that impact staff as they attempt to do the work of caring for their patients. It is apparent from the findings in this study that taking a CST perspective that underlines ID can help researchers to explore and engage with the complexities and contexts of people’s experiences. CST is a means for creating different ways of knowing and understanding relationships, which are characterized by difference (Wilson-Thomas, 1995).

There is a temptation to name, label and keep people in their place. They are often lumped together as an indistinguishable whole, even though each individual has a subjective reality. ID attempts to not only describe thematic patterns and commonalities but to also account for individual variations (Thorne et al., 2004). It is important that the naming and labeling that is an intrinsic piece of qualitative research is not allowed to oversimplify our understandings. CST has the potential to advance practices toward socially relevant and progressive emancipatory possibilities (Browne, 2000), but care must be taken to engage in a reflective process that ensures that prevailing norms and accepted truths or realities (such as inadequate physical spaces), are challenged. It is important to attend to what is most important, which is the social, political and structural conditions that impact on the lives and experiences of both patients and caregivers.

Front line nurses often have difficulty appreciating how research can affect and influence their working lives. There is a disparity between what is known and what is actually done to solve basic clinical problems. It is important to make a connection between the researcher’s worldview, the experiences of the staff and the sociopolitical norms that frame a workplace. It is crucial to report the findings of the study in ways that shift oppressive structures and policies. Frontline staff need to be engaged in understanding and shaping their work life. Taking a CST perspective to describe the issues of power and describing the clinical outcomes of this power imbalance with an ID methodology is one way of ensuring that frontline staff understand what they confront in their everyday working lives.

## Conclusion

It can be a difficult task to determine the direction of research. Formulating a plan, selecting a methodology, determining a prospective sample and deciding on an analytic strategy can all be fraught. It is important to connect research methodologies with theoretical concerns and commitments (Lather, 1986). In this paper we indicate that there is a clear and fruitful relationship between CST and ID. When used together, there is the potential for a different form of knowledge, a knowledge that exposes how participants experience their realities to make visible the discourses and structures that influence it. CST has the potential to ensure that an ID methodology allows for a thorough examination of the experiences of health care staff. Outlining key findings from a research study, we have made visible the fit between a CST approach and an ID study design.

## References

[bibr1-23333936231211462] Brathwaite B. (2018). Black, Asian and minority ethnic female nurses: Colonialism, power and racism. The British Journal of Nursing, 27(5), 254–258. 10.12968/bjon.2018.27.5.254 29517323

[bibr2-23333936231211462] Browne A. J. (2000). The potential contributions of critical social theory to nursing science. The Canadian Journal of Nursing Research, 32(2), 35–55. 11151569

[bibr3-23333936231211462] Connelly L. M. (2014). Use of theoretical frameworks in research. Medsurg Nursing, 23(3), 187–188. 25137796

[bibr4-23333936231211462] Cresswell J. W. (Ed.). (2006). Qualitative inquiry and research design: Choosing among five approaches (2nd ed.). Sage.

[bibr5-23333936231211462] Crocker J. Major B. (1989). Social stigma and self-esteem: The self-protective properties of stigma. Psychological Review, 96(4), 608–630. 10.1037/0033-295x.96.4.608

[bibr6-23333936231211462] Freire P. (1998). Pedagogy of the heart. In Freire A. Macedo D. (Eds.), The Paulo Freire reader (pp. 265–282). Continuum.

[bibr7-23333936231211462] Friere P. (1970). Pedagogy of the oppressed. Seabury.

[bibr8-23333936231211462] Fulton Y. (1997). Nurses’ views on empowerment: A critical social theory perspective. Journal of Advanced Nursing, 26, 529–536. 10.1046/j.1365-2648.1997.t01-13-00999.x 9378874

[bibr9-23333936231211462] Gieryn T. F. (2000). A space for place in sociology. The Annual Review of Sociology, 26, 463–496.

[bibr10-23333936231211462] Glass R. D. (2001). On Paulo Freire’s philosophy of praxis and the foundations of liberation education. Educational Researcher, 30(2), 15–25.

[bibr11-23333936231211462] Hall J. M. (1999). Marginalization revisited: Critical, postmodern and liberation perspectives. Advances in Nursing Science, 22(2), 88–102. 10.1097/00012272-199912000-00009 10634190

[bibr12-23333936231211462] Hart C. (2015). The elephant in the room: Nursing and nursing power on an interprofessional team. The Journal of Continuing Education in Nursing, 46(8), 349–355. 10.3928/00220124-20150721-01 26247656

[bibr13-23333936231211462] Hopkins V. Harvey E. O’Brien K. (2004). Advocacy for drug users. Scottish Executive Effective Interventions Unit.

[bibr14-23333936231211462] Hunt M. R. (2009). Strengths and challenges in the use of interpretive description: Reflections arising from a study of the moral experience of health professionals in humanitarian work. Qualitative Health Research, 19(9), 1284–1292. 10.1177/1049732309344612 19690208

[bibr15-23333936231211462] Hutchinson M. Jackson D. (2015). The construction and legitimation of workplace bullying in the public sector: Insight into power dynamics and organisational failures in health and social care. Nursing Inquiry, 22(1), 13–26. 10.1111/nin.12077 25131347

[bibr16-23333936231211462] Kasule G. W. (2015). Impact of work environment on academic staff job performance: Case of a Uganda university. International Journal of Advances in Management and Economics, 4(4), 95–103.

[bibr17-23333936231211462] Kitchin R. (1998). ‘Out of place’, knowing one’s place’: Space, power and the exclusion of disabled people. Disability & Society, 13(3), 343–356. 10.1080/09687599826678

[bibr18-23333936231211462] Lantholf J. P. Poehner M. P. (2014). Sociocultural theory and the pedagogical imperative in L2 education. Routledge.

[bibr19-23333936231211462] Lather P. (1986). Research as praxis. Harvard Educational Review, 56(3), 257–278. 10.17763/haer.56.3.bj2h231877069482

[bibr20-23333936231211462] Leonardo Z. (2004). Critical social theory and transformative knowledge: The functions of criticism in quality education. Educational Researcher, 33(6), 11–18. 10.3102/0013189x033006011

[bibr21-23333936231211462] Lincoln Y. S. Guba E. G. (1985). Naturalistic inquiry. Sage.

[bibr22-23333936231211462] Liu F. Harrell S. (2015). Exploring epistemological implications of the yin-yang theory. The Journal of Theory Construction and Testing, 19(1), 15–20.

[bibr23-23333936231211462] Lloyd-Walker B. Walker D. (2015). Moving from hunches to a research topic: Salient literature and research methods. In Pasain B. (Ed.), From designs, methods and practices for research of project management (pp. 119–129). Routledge.

[bibr24-23333936231211462] Lupton D. (1992). Discourse analysis: A new methodology for understanding the ideologies of health and illness. Australian Journal of Public Health, 16, 145–150. 10.1111/j.1753-6405.1992.tb00043.x 1391155

[bibr25-23333936231211462] Manias E. Street A. (2000). Possibilities for critical social theory and Foucault’s work: A toolbox approach. Nursing Inquiry, 7, 50–60. 10.1046/j.1440-1800.2000.00048.x 11022535

[bibr26-23333936231211462] Mantzoukas S. Jasper M. A. (2004). Reflective practice and daily ward reality: A covert power game. Journal of Clinical Nursing, 13(8), 925–933. 10.1111/j.1365-702.2004.01008.x 15533098

[bibr27-23333936231211462] McCall J. (2018). The Crosstown Clinic: The experience of staff and patient in a heroin assisted treatment program in Canada [Doctoral Dissertation, University of Alberta]. ProQuest Dissertations & Theses Global.

[bibr28-23333936231211462] McCall J. Phillips J. C. Estafan A. Caine V. (2019). Exploring the experiences of staff working at an opiate assisted treatment clinic: An interpretive descriptive study. Applied Nursing Research: ANR, 45, 45–51. 10.1016/j.apnr.2018.12.003 30683250

[bibr29-23333936231211462] McCall J. Phillips J. C. Estafan A. Caine V. (2020). The patients have a story to tell: Informed consent for people who use illicit opiates. Nursing Ethics, 27(3), 666–672. 10.1177/0969733020901814 32101076

[bibr30-23333936231211462] McLaughlin N. (1999). Origin myths in the social sciences: Fromm, the Frankfurt School, and the emergence of critical theory. The Canadian Journal of Sociology, 24(1), 109–139. 10.2307/3341480

[bibr31-23333936231211462] Ocean M. Montgomery R. Jamison Z. Hicks K. Thorne S. (2022). Exploring the expansive properties of interpretive description: An invitation to anti-oppressive researchers. International Journal of Qualitative Methods, 21, 1–14. 10.1177/16094069221103665

[bibr32-23333936231211462] O’Byrne P. (2019). HIV PEP and nursing scholarship: A review of critical theory and social justice. Witness: The Canadian Journal of Critical Nursing Discourse, 1(1), 28–38. 10.25071/2291-5796.20

[bibr33-23333936231211462] Phillips R. E. (2009). Nurses’ experience with HIV stigma in five African countries [Doctoral Dissertation, University of California]. ProQuest Dissertations & Theses Global.

[bibr34-23333936231211462] Poteat T. German D. Kerrigan D. (2013). Managing uncertainty: A grounded theory of stigma in transgender health care encounters. Social Science & Medicine (1967), 84, 22–29. 10.1016/j.socscimed.2013.02.019 23517700

[bibr35-23333936231211462] Putnam H. (1999). The threefold cord: Mind, body and world. Columbia University Press.

[bibr36-23333936231211462] Sandelowski M. (2010). What’s in a name? Qualitative description revisited. Research in Nursing & Health, 33, 77–84. 10.1002/nur.20362 20014004

[bibr37-23333936231211462] Smith L. J. (2008). How ethical is ethical research? Recruiting marginalized, vulnerable groups into health services research. Journal of Advanced Nursing, 62(2), 248–257. 10.1111/j.1365-2648.2007.04567.x 18394037

[bibr38-23333936231211462] Soja E. W. (2003). Postmodern geographies: The reassertion of space in critical social theory. Verso.

[bibr39-23333936231211462] Sonn C. C. Quayle A. F. (2013). Developing praxis: Mobilising Critical Race Theory in community cultural development. Journal of Community & Applied Social Psychology, 23(5), 435–448. 10.1002/casp.2145

[bibr40-23333936231211462] Stevens P. E. Hall J. M. (1992). Applying critical theories to nursing in communities. Public Health Nursing, 9(1), 2–9. 10.1111/j.1525-1446.1992.tb00065.x 1565599

[bibr41-23333936231211462] Sumner J. Danielson E. (2007). Critical social theory as a means of analysis for caring in nursing. International Journal for Human Caring, 11(1), 30–37.

[bibr42-23333936231211462] Thorne S. (1997). The art (and science) of critiquing qualitative research. In Morse J. (Ed.), Completing a qualitative project: Discussions and dialogue (pp. 117–132). Sage.

[bibr43-23333936231211462] Thorne S. (2008). Interpretive description. Left Coast Press.

[bibr44-23333936231211462] Thorne S. Kirkham S. R. O’Flynn-Magee K. (2004). The analytic challenge in interpretive description. International Journal of Qualitative Methods, 3(1), 1–11. 10.1177/160940690400300101

[bibr45-23333936231211462] Vinz S. George T. (2022). What is a theoretical framework? Guide to organizing. https://www.scribbr.com/

[bibr46-23333936231211462] Wilson-Thomas L. (1995). Applying critical social theory in nursing education to bridge the gap between theory, research and practice. Journal of Advanced Nursing, 21, 568–575. 10.1111/j.1365-2648.1995.tb02742.x 7745213

[bibr47-23333936231211462] Yang L. H. Kleinman A. Link B. G. Phelan J. C. Lee S. Good B. (2007). Culture and stigma: Adding moral experience to stigma theory. Social Science & Medicine (1967), 64(7), 1524–1535. 10.1016/j.socscimed.2006.11.013 17188411

